# Epidemiology of Epidemic Ebola Virus Disease in Conakry and Surrounding Prefectures, Guinea, 2014–2015

**DOI:** 10.3201/eid2202.151304

**Published:** 2016-02

**Authors:** Adriana Rico, Debra Brody, Fátima Coronado, Marc Rondy, Lena Fiebig, Andrea Carcelen, Varough M. Deyde, Samuel Mesfin, Kyla D. Retzer, Pepe Bilivogui, Sakoba Keita, Benjamin A. Dahl

**Affiliations:** Centers for Disease Control and Prevention, Atlanta, Georgia, USA (A. Rico, F. Coronado, A. Carcelen, B.A. Dahl);; Centers for Disease Control and Prevention, Hyattsville, Maryland, USA (D. Brody);; EpiConcept, Paris, France (M. Rondy);; World Health Organization Ebola Response Team, Conakry, Guinea (M. Rondy, L. Fiebig, S. Mesfin);; Robert Koch Institute, Berlin, Germany (L. Fiebig);; Centers for Disease Control and Prevention, Pretoria, South Africa (V.M. Deyde);; Centers for Disease Control and Prevention, Denver, Colorado, USA (K.D. Retzer);; Ministry of Health, Conakry (P. Bilivogui, S. Keita)

**Keywords:** Ebola Virus Disease, epidemic, control, Guinea, viruses, Africa, Ebola

## Abstract

In 2014, Ebola virus disease (EVD) in West Africa was first reported during March in 3 southeastern prefectures in Guinea; from there, the disease rapidly spread across West Africa. We describe the epidemiology of EVD cases reported in Guinea’s capital, Conakry, and 4 surrounding prefectures (Coyah, Dubreka, Forecariah, and Kindia), encompassing a full year of the epidemic. A total of 1,355 EVD cases, representing ≈40% of cases reported in Guinea, originated from these areas. Overall, Forecariah had the highest cumulative incidence (4× higher than that in Conakry). Case-fatality percentage ranged from 40% in Conakry to 60% in Kindia. Cumulative incidence was slightly higher among male than female residents, although incidences by prefecture and commune differed by sex. Over the course of the year, Conakry and neighboring prefectures became the EVD epicenter in Guinea.

## Abstract 2

The capital and neighboring areas remain a focal point of transmission, requiring continued public health vigilance.

Ebola virus disease (EVD) in West Africa was first reported during early March 2014 in Guinea’s 3 southeastern prefectures (Gueckedou, Macenta, and Kissidougou), which border Liberia and Sierra Leone; however, retrospective investigations indicate Ebola virus (EBOV) transmission might have occurred in Guinea since December 2013 (*1*–*4*). On March 27, 2014, EVD was reported in Conakry (population 1,667,864), the capital of and largest city in Guinea (*1*,*5*). EBOV rapidly spread through much of Guinea, where it was reported in 32 of 34 prefectures, and to Liberia and Sierra Leone, causing the largest EVD epidemic since EBOV was discovered in 1976 (*2*,*3*,*6*). As of November 1, 2015, West Africa reported >28,000 EVD cases, of which >3,800 (including >2,500 deaths) were reported from Guinea; (*7*). The presence of EVD in Conakry led the Guinea Ministry of Health (MoH) to request assistance from the US Centers for Disease Control and Prevention, the World Health Organization (WHO), Médecins Sans Frontières, and other partners to establish a systematic disease-surveillance process and to implement control measures nationwide. Here we characterize EVD cases in Conakry and the 4 surrounding prefectures, which together became the epicenter of the EVD epidemic in.

## Methods

We conducted a descriptive analysis of data reported in the Epi Info Viral Hemorrhagic Fever Application (Epi Info VHF, http://epiinfovhf.codeplex.com/), software used to maintain the Guinea MoH national case database. Patient-specific data (i.e., demographic, clinical, epidemiologic, and laboratory information) were collected by using a standardized EVD case notification form. Demographic information (age, sex, and residence) was obtained from the standardized notification forms completed during patient admission to an Ebola treatment center (ETC) or at the corpse collection for persons who died outside of an ETC or hospital (community deaths). Final vital outcome status for patients admitted to an ETC was obtained from ETC line listing data; all data were updated in Epi Info VHF.

EVD cases were classified into 1 of 3 WHO case definitions: suspected, probable, or laboratory-confirmed cases. A suspected case was defined as disease in a living person with 1) a history of contact with a person who had laboratory-confirmed or probable EVD and 2) who had unexplained bleeding or sudden onset of high fever or >3 of the following signs and symptoms: headache, anorexia/loss of appetite, lethargy, aching muscles or joints, breathing difficulties, vomiting, diarrhea, stomach pain, difficulty swallowing, and hiccups. Probable cases were defined as disease in deceased persons who had an epidemiologic association with EVD but no laboratory testing. Laboratory-confirmed cases were defined as cases in any persons, dead or alive, who had laboratory-confirmed EVD (*8*,*9*). Laboratory confirmation of EVD cases was made on the basis of positive real-time reverse transcription PCR results or, for samples tested >10 days after symptom onset and for PCR-negative samples, on the basis of positive serologic testing results. Only laboratory-confirmed and probable cases are described in this report because suspected cases had already been reclassified at the time of this analysis.

Our analysis included cases reported in Epi Info VHF during January 1, 2014–March 29, 2015 (epidemiologic week 1, 2014, to epidemiologic week 13, 2015). All persons whose place of residence was listed as Conakry, including its 5 communes (Dixinn, Kaloum, Matam, Matoto, and Ratoma), or as 1 of the 4 surrounding prefectures (Coyah, Dubreka, Forecariah, and Kindia) were included in the analysis. Epidemiologic weeks were in accordance with those designated by in-country situation reports. For numerators for cumulative incidence, we used all laboratory-confirmed and probable cases, by commune and prefecture. For denominators, we used preliminary 2014 population data obtained from the Guinea National Statistics Institute, Ministry of Planning (*10*). To remain consistent with WHO reporting, we calculated the case-fatality percentage by using the number of laboratory-confirmed deaths in ETCs as the numerator and the number of laboratory-confirmed cases for which final status was known, excluding community deaths, as the denominator. The percentage of laboratory-confirmed community deaths was determined by dividing the number of laboratory-confirmed community deaths by the total number of laboratory-confirmed cases. These surveillance data were collected and used for public health practice purposes to control the epidemic, not for human subject research.

## Results

During January 1, 2014–March 29, 2015, a total of 553 EVD cases were reported in Conakry, and an additional 802 were reported in the 4 surrounding prefectures. Among these 1,355 cases, a total of 1,226 (90%) were laboratory-confirmed and 129 (10%) were probable cases. The median age of persons with EVD was 30 years (lower and upper quartiles 20 and 45 years, respectively); 283 (21%) infected persons were <18 years of age, and 671 (50%) were female. The most commonly reported signs and symptoms during the first visit to an ETC were fever (96%), fatigue (96%), and anorexia (86%). Records indicated that 118 (9%) EVD cases were in healthcare workers. A total of 817 (60%) infected persons died; of these, 259 (21%) died in the community (Tables 1, 2). The number of community deaths per epidemiologic week fluctuated from 0 to 27.

**Table 1:** Ebola virus disease cases by prefecture and sex in Conakry, the capital city, and surrounding prefectures, Guinea, January 1, 2014–March 29, 2015*

Location	No. (%) cases by classification			No. (%) cases, no. cases/100,000 persons				Median age, y (Q1, Q3)	% ETC case-fatality, (95% CI)†	No. (%) community deaths‡
	Laboratory confirmed	Probable								
				Total	Men	Women				
Conakry	519 (42)	34 (26)		553 (41), 33.2	307 (45), 36.6	246 (37), 29.7		30 (22, 44)	40 (35–45)	93 (18)
Coyah	229 (19)	7 (6)		236 (17), 89.3	112 (16), 85.5	124 (18), 93.1		30 (20, 43)	47 (40–55)	44 (19)
Dubreka	115 (9)	8 (6)		123 (9), 37.5	65 (9), 40.3	58 (9), 34.7		30 (18, 40)	46 (35–58)	32 (28)
Forecariah	290 (24)	45 (35)		335 (25), 136.9	155 (23), 132.2	180 (27), 141.3		30 (18, 45)	53 (46–60)	78 (27)
Kindia	73 (6)	35 (27)		108 (8), 24.6	45 (7), 21.2	63 (9), 27.8		35 (22, 50)	60 (47–73)	12 (16)
Total	1,226 (100)	129 (100)		1,355 (100), 46.0	684 (100), 46.8	671 (100), 45.3		30 (20, 45)	46 (43–49)	259 (21)

*Data were obtained from the Guinea Ministry of Health national case database (Epi Info Viral Hemorrhagic Fever Application, http://epiinfovhf.codeplex.com/). ETC, Ebola treatment center; Q1 and Q3, lower and upper quartiles, respectively. †Case fatality = ETC deaths/(laboratory-confirmed cases with final status known – community deaths); see Table 2. ‡Percent community deaths = community deaths/laboratory-confirmed cases; see Table 2.

**Table 2:** Ebola virus disease cases and associated deaths Conakry, the capital city, and surrounding prefectures, Guinea, January 1, 2014–March 29, 2015*

Location	No. cases by classification							
	Total	Probable	Laboratory-confirmed	Laboratory confirmed with known final status		No. (%) deaths		
						ETC	Community	Total†
Prefectures								
Conakry	553	34	519	513		168	93	295 (53)
Coyah	236	7	229	221		84	44	135 (57)
Dubreka	123	8	115	110		36	32	76 (62)
Forecariah	335	45	290	277		106	78	229 (68)
Kindia	108	35	73	70		35	12	82 (76)
Total	1,355	129	1,226	1,191		429	259	817(60)
Conakry commune‡								
Dixinn	59	2	57	56		15	11	28 (47)
Kaloum	55	7	48	48		9	8	24 (44)
Matam	61	6	55	53		19	9	34 (56)
Matoto	198	13	185	185		62	43	118 (60)
Ratoma	145	4	141	140		51	20	75 (52)

*Data were obtained from the Guinea Ministry of Health national case database (Epi Info Viral Hemorrhagic Fever Application, http://epiinfovhf.codeplex.com/). ETC, Ebola treatment center.  †Probable plus ETC plus community deaths divided by all cases. ‡Counts do not sum because 35 cases were missing commune information.

**Figure 1:**
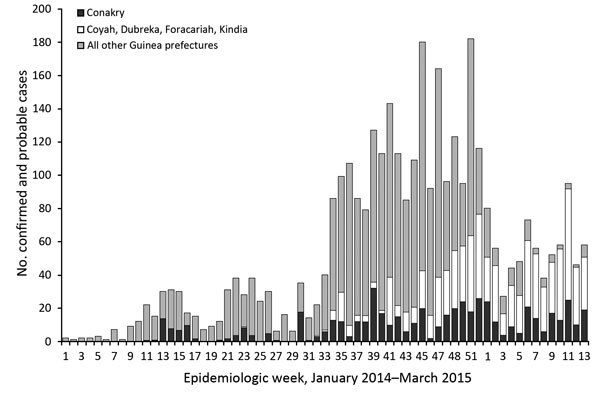
Ebola virus disease cases in Conakry, the capital city; 4 surrounding prefectures; and all remaining prefectures, Guinea, January 1, 2014–March 29, 2015. Data were obtained from the Guinea Ministry of Health national case database (Epi Info Viral Hemorrhagic Fever Application). Epidemiologic week 52 ended on December 27, 2014.

The first laboratory-confirmed EVD case in Conakry was reported in late March 2014 (epidemiologic week 11), approximately 3 months after cases were identified in Guinea (Figure 1). During March–September 2014, the number of weekly EVD cases reported in Conakry ranged from 0 to 18 (Figure 1). During early October (epidemiologic week 39), the number of weekly reported cases in Conakry peaked at 32. Beginning mid-January 2015 (epidemiologic week 3, 2015), weekly EVD cases for all Guinea prefectures briefly declined, and most of the weekly cases reported at that time and up until March 2015 were among Conakry residents and persons residing in the 4 neighboring prefectures (Figure 1).

**Figure 2:**
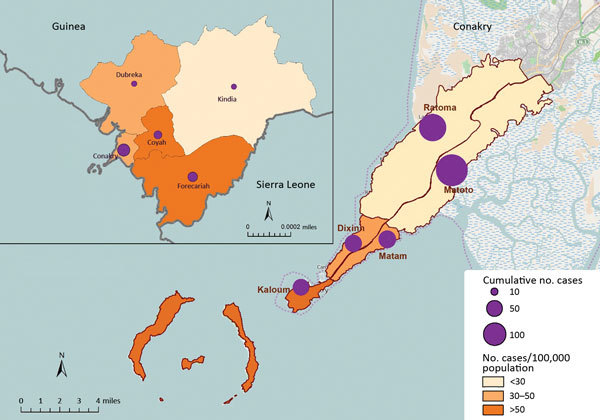
Cumulative incidence of Ebola virus disease cases in Conakry, the capital city, and 4 surrounding prefectures, Guinea, January 1, 2015–March 29, 2015. Data were obtained from the Guinea Ministry of Health national case database (Epi Info Viral Hemorrhagic Fever Application, http://epiinfovhf.codeplex.com/). A total of 35 cases were missing commune information and are not represented in the figure. Inset shows locations of prefectures in Guinea; larger map shows locations of communes in Conakry.

During the study period, the overall number of EVD cases per 100,000 persons was 33.2 in Conakry, 89.3 in Coyah, 37.5 in Dubreka, 136.9 in Forecariah, and 24.6 in Kindia (Table 1; Figure 2). Cumulative incidence was slightly higher among male (46.8 cases/100,000 persons) than female (45.3 cases/100,000 persons) residents. Furthermore, incidence varied by sex in prefectures; incidence was higher among female residents in Coyah, Forecariah, and Kindia (Table 1). Excluding community deaths, the case-fatality percentage among EVD-infected persons ranged from 40% (95% CI 35%–45%) in Conakry to 60% (95% CI 47%–73%) in Kindia. Among all cases, community deaths were highest for residents of Dubreka (28%) and Forecariah (27%).

Among the 5 communes in Conakry, Kaloum, the smallest by population, had the highest overall incidence (87.8 cases/100,000 persons) but the lowest case-fatality percentage (23%) (Table 3). Ratoma, the second most populated commune, had the lowest overall incidence (22.2 cases/100,000 persons) but a case-fatality percentage of 43%. Kaloum was the only commune that had a higher incidence of EVD among female (97.9 cases/100,000 persons) than male (77.4 cases/100,000 persons) residents. The percentage of EVD community deaths was highest in Matoto (23%).

**Table 3:** Ebola virus disease cases by Conakry, the capital city, and its 5 communes, Guinea, January 1, 2014– March 29, 2015*

Location, commune	No. (%) cases by classification			No. (%) cases, no. cases/100,000 persons				Median age, y (Q1, Q3)	% ETC case-fatality (95% CI)†	No.(%) community deaths‡
	Laboratory confirmed	Probable								
				Total	Men	Women				
Conakry§	519 (100)	34 (100)		553 (100) 33.2	307 (100) 36.6	246 (100) 29.7		30 (22,44)	40 (35–45)	93 (18)
Dixinn	57 (12)	2 (6)		59 (11) 42.9	35 (12) 50.2	24 (10) 35.5		29 (18,44)	33 (20–49)	11 (19)
Kaloum	48 (10)	7 (22)		55 (11) 87.8	24 (9) 77.4	31 (13) 97.9		30 (19,41)	23 (11–38)	8 (17)
Matam	55 (11)	6 (19)		61 (12) 42.5	31 (11) 43.4	30 (13) 41.5		35 (26,45)	43 (28–59)	9 (16)
Matoto	185 (38)	13 (41)		198 (38) 29.5	115 (40) 34.1	83 (36) 24.9		32 (22,45)	44 (35–52)	43 (23)
Ratoma	141 (29)	4 (12)		145 (28) 22.2	79 (28) 23.9	66 (28) 20.4		30 (23,40)	43 (34–52)	20 (14)

*Data were obtained from the Guinea Ministry of Health national case database (Epi Info Viral Hemorrhagic Fever Application, http://epiinfovhf.codeplex.com/). ETC, Ebola treatment center; Q1 and Q3, lower and upper quartiles, respectively. †Case fatality = ETC deaths/(laboratory-confirmed cases with final status known – community deaths); see Table 2. ‡Percent community deaths = community deaths/laboratory-confirmed cases; see Table 2. §Counts do not sum because 35 cases were missing commune information.

## Discussion

EVD transmission first occurred during March 2014 in Conakry, the capital and largest city in Guinea. During the weeks that followed, reported EVD cases in the capital remained low, but virus transmission continued in the city. Sustained transmission was attributed to the continued refusal by a limited number of families to accept clinical intervention and isolation (*11*). However, in December 2014 (epidemiologic week 52), the epidemic peaked in Conakry and the 4 surrounding prefectures (Figure 1); at that time, EVD cases in Conakry, Coyah, Dubreka, Kindia, and Forecariah represented most cases in Guinea. This shift of the EVD outbreak from other parts of Guinea to Conakry, with its population of 1.7 million persons, was a landmark event during the epidemic, and implementation of targeted control measures fortunately prevented substantial outbreak amplification (*11*). Cenciarelli et al. (*12*) suggest that Guinea had better EVD management, treatment, and laboratory support than the other affected countries, resulting in Guinea having a slower rise in cases. Conakry and the 4 surrounding prefectures remained a principal focus of the outbreak in Guinea. From the beginning of the epidemic in the capital, a total of >550 EVD cases were reported from Conakry and >800 were reported from the 4 surrounding prefectures. Together, these areas accounted for ≈40% of the total number of cases in Guinea during the study period.

The overall incidence of EVD cases varied by prefecture; Forecariah, a prefecture bordering Sierra Leone, had an incidence 4 times higher than that of Conakry. Kaloum, the smallest (by population size) commune in Conakry, had the highest overall incidence and highest incidence among female residents. Overall, the cumulative incidence of EVD cases was slightly higher for male than female residents; however, the incidence among female residents was higher than that among male residents in 3 prefectures (Coyah, Forecariah, and Kindia) and 1 commune (Kaloum). A United Nations report suggests that this difference in incidence by sex may be attributed to the role of women as primary frontline caregivers for sick persons, putting them at a higher risk for exposure to EBOV (*13*).

Continued EVD transmission in the Conakry area is attributed to multiple factors, including community and family transmission, high mobility of EVD patients to and from Conakry and neighboring prefectures, and localized resistance to EVD interventions (*11*). Case investigations have shown that residents of Conakry often have relatives in other prefectures whom they visit (and vice versa) (*11*). In addition, patients with EVD-like symptoms travel to Conakry to seek treatment and improved healthcare services (*6*). On arrival at ETCs, these patients, compared with those who do not have to travel for care, can be further along in the disease course and have high virus loads, increasing the risk for exposure of healthcare personnel, relatives, and other community members before hospitalization and isolation. In addition, certain patients might have been hospitalized or otherwise cared for outside of ETCs, causing a potential for outbreak amplification and continuation of the EVD transmission chain in the community, as demonstrated in previously reported cases (*2*). We showed that the case-fatality percentage in Conakry was lower than that in the surrounding prefectures, possibly reflecting differences in resources and case management. Conakry has many public and private hospitals and clinics, including Donka Hospital, the site of Conakry’s first ETC (*2*). Additional challenges to reducing disease transmission in the capital area included initial limited awareness and acceptance of the disease, fear and mistrust, and stigma associated with the disease (*6*,*14*).

Although EVD surveillance via Epi Info VHF in Conakry and throughout Guinea was constantly updated to accurately capture correct case information, the database captures only what is reported. For example, certain variables (e.g., clinical data) are often incomplete. Furthermore, a study from Barry et al. (*6*) indicated a general underreporting of EVD cases because certain patients never seek medical care at an ETC. Therefore, EVD incidence in Conakry and the 4 surrounding prefectures is probably higher than we report (*6*). Enhancements to the primary data collection systems at the national and prefecture levels have been ongoing in Guinea. Starting in early 2015, weekly situation reports included detailed prefecture assessments with commune-level analyses, indicators to measure the number and severity of all security incidents (e.g., violent threats toward public health professionals), and situations of refusal to cooperate (e.g., failure to disclose names of contacts). The integration of transmission data chains with primary case notification records enables the identification of new cases from known contacts and unknown chains of transmission.

Our findings are subject to limitations. First, notable underreporting of probable cases is indicated by a higher number of investigated burials included in aggregated country daily reports produced by the Guinea MoH with the assistance of WHO and other partners. Underreporting of probable cases might be unevenly distributed across the country and thus affect comparisons with cumulative case numbers across prefectures. Efforts to retrospectively complete case-based notification of probable cases were strengthened starting at the end of 2014. Second, the information (e.g., ascertainment of professions, including healthcare workers) across these settings is incomplete and may involve information bias. Ongoing data quality assessments are in place and focus on key variables, including case definition category, final vital status, and prefecture of residence. Despite these limitations, comparisons with aggregated figures from situation reports and case-based data from the national database indicate that Guinea’s Epi Info VHF data represent the history of this EVD epidemic (*7*).

In summary, in late December 2014, during the first year of the Ebola epidemic, weekly cases in Conakry and the 4 surrounding prefectures surpassed reported cases from all other Guinea prefectures. To date, these areas have remained a focal point of disease transmission. High mobility within Conakry and surrounding prefectures is common, and, thus, contact tracing and transmission chain tracking are challenging. Because of these factors, the daily information exchange regarding cases and contacts among epidemiologists and contact tracers working in Conakry and the surrounding prefectures is essential. Finally, improving surveillance efforts at the commune level to identify where more prevention and effective communication measures are needed is critical.
